# Acyl Carrier Protein 3 Is Involved in Oxidative Stress Response in *Pseudomonas aeruginosa*

**DOI:** 10.3389/fmicb.2018.02244

**Published:** 2018-09-20

**Authors:** Wei Chen, Bo Wang, Jordon D. Gruber, Yong-Mei Zhang, Christopher Davies

**Affiliations:** ^1^Clinical Research Center, The Second Affiliated Hospital of the Southeast University, Nanjing, China; ^2^Department of Biochemistry and Molecular Biology, Medical University of South Carolina, Charleston, SC, United States; ^3^Institute of Medicinal Biotechnology, Chinese Academy of Medical Sciences and Peking Union Medical College, Beijing, China

**Keywords:** acyl carrier protein, *P. aeruginosa*, virulence, catalase, oxidative stress

## Abstract

The human opportunistic pathogen *Pseudomonas aeruginosa* expresses three acyl carrier proteins (ACPs): AcpP, Acp1, and Acp3. The function of AcpP in membrane fatty acid synthesis (FAS) was confirmed recently, but the physiological roles of Acp1 and Acp3 remain unclear. To address this, we investigated the physiological role of Acp3 in *P. aeruginosa*. We found that expression of Acp3 dramatically increases in the log phase of cell growth and that its transcription is under the control of the QS regulators LasR and RhlR. Deletion of *acp3* from *P. aeruginosa strain* PAO1 results in thicker biofilm formation, increased resistance of the strain to hydrogen peroxide, and higher persistence in a mouse infection model. Tandem affinity purification (TAP) experiments revealed several novel protein-binding partners of Acp3, including KatA, the major catalase in *P. aeruginosa*. Acp3 was found to repress the catalase activity of KatA and, consistent with inhibition by Acp3, less reactive oxygen species are present in the *acp3* deletion strain. Overall, our study reveals that Acp3 has a distinct function from that of the canonical AcpP and may be involved in the oxidative stress response.

## Introduction

Acyl carrier proteins (ACPs) function in the synthesis of fatty acids, polyketides, and non-ribosomal peptides. In addition to the housekeeping ACP used for Type II fatty acid synthesis (FAS), some bacteria contain specialized variants of ACPs (Geiger et al., 1991; Brozek et al., 1996; Epple et al., 1998; Geiger and Lopez-Lara, 2002; Haag et al., 2009; Ramos-Vega et al., 2009; Chan and Vogel, 2010). Some of these divert intermediates of Type II FAS (FAS) to synthesize molecules like lipid A or hemolysin, whereas others are involved in distinct processes such as polyketide synthesis (Byers and Gong, 2007; Chan and Vogel, 2010). *Rhizobia*, for example, expresses four ACPs (AcpP, NodF, AcpXL, and RkpF), where AcpP is the canonical ACP for FAS and the other three are used for synthesis of cell-surface molecules that facilitate the establishment of symbiosis with their legume hosts (Geiger and Lopez-Lara, 2002). In addition, it was reported recently that in *Salmonella enterica*, an ACP homologue called IacP acylates a type 3 secretion system (T3SS) translocon protein, SipB, leading to insertion of SipB into host-cell membranes (Viala et al., 2017).

By contrast, *Escherichia coli* encodes only a single and essential ACP (De Lay and Cronan, 2007). In addition to functioning in Type II FAS, however, it also acts in several other systems. For instance, the protein interacts with SpoT to switch the activity of SpoT from (p)ppGpp degradation to (p)ppGpp synthesis during conditions of fatty acid starvation (Battesti and Bouveret, 2006; Angelini et al., 2012) and also forms a complex with the STAS domain of a SLC26 family anion transporter YchM, linking bicarbonate transport with fatty acid metabolism (Babu et al., 2010). In addition, *E. coli* ACP plays an essential role in the biosynthesis of membrane-derived oligosaccharides (MDOs), which are important for osmotic regulation (Therisod et al., 1986). Interestingly, the 4-phosphopantetheine moiety of ACP is not required in the MDO transglucosylation function, a clear difference from ACP’s role in fatty acid biosynthesis (Therisod and Kennedy, 1987).

*Pseudomonas aeruginosa* is an opportunistic pathogen that causes life-threatening nosocomial infections and is of particular concern for immunocompromised and cystic fibrosis (CF) patients (Govan and Deretic, 1996). The *P. aeruginosa* genome contains three putative *acp* genes, annotated as PA2966 (*acpP*), PA1869 (*acp1*), and PA3334 (*acp3*). Each contains a conserved serine that is presumed to be the site of 4-phosphopantetheinylation (Raychaudhuri et al., 2005). Recently, AcpP was confirmed as the essential ACP used for membrane FAS, and deletion of *acp1* and *acp3* had minimal effect on cell growth and *in vitro* virulence production (Ma et al., 2017). Previous studies have showed that *acp1* and *acp3* expression is regulated by quorum sensing (QS) (Hentzer et al., 2003; Schuster et al., 2003; Wagner et al., 2003; Chugani et al., 2012) and *acp3* is highly induced during infection (Turner et al., 2014). In particular, the 18-fold increase in expression of *acp3* in an acute infection model and 30-fold increase in a chronic infection model strongly suggests a key role for Acp3 in pathogenicity (Turner et al., 2014). Beyond these indicators, however, the physiological functions of Acp1 and Acp3 in *P. aeruginosa* remain unclear.

In this work, we examined the specific physiological role of Acp3 in *P. aeruginosa via* a combination of transcriptional profiling, western blotting, measuring *in vitro* and *in vivo* virulence, and identification of protein-binding partners. We found that *acp3* is regulated by the QS system and that expression of Acp3 peaks during the early stationary phase of cell growth. Deletion of *acp3* increases biofilm formation, increased resistance of the strain to H_2_O_2_ and higher persistence in the mouse lung. We also found that Acp3 binds and represses the activity of the catalase, KatA. Overall, the data suggest a specialized function for Acp3 involving oxidative response mediated by KatA.

## Materials and Methods

### Bacterial Strains, Plasmids, Primers, and Growth Conditions

Bacterial strains and plasmids used in this study are listed in **Table 1**. Strain stocks were maintained at -80°C in 10% glycerol. Bacteria were streaked on fresh plates before each experiment. *P. aeruginosa* and *E. coli* strains were cultured at 37°C in Miller’s Luria broth (LB) medium (Sigma-Aldrich) unless specified otherwise. *Pseudomonas* Isolation Agar (PIA) was used for selection of *P. aeruginosa*. Plasmids used for gene knockout were constructed using the described method (Choi and Schweizer, 2005). Primers used in this study are listed in **Supplementary Table S1**.

**Table 1:** Strains and plasmids used in this study.

Strain or plasmid	Relevant genotype or phenotype	Reference
***E. coli* strains**		
JM109	*F′ (traD36, proAB + lacI^q^ lacZ ΔM15 endA1 recA1 hsdR17(r_k_*^-^*, m_k_^+^) mcrA supE44 λ gyrA96 relA1 Δ(lacproAB)*	Promega
CY1861	*ΔacpP, fabF::Cml, pCY765*	Volkmann et al., 2010
SM10 lpir	*thi-1 thr leu tonA lacY supE recA::RP4-2-Tc::Mu Km^r^ lpir*	de Lorenzo and Timmis, 1994
XL-1 blue	*recA1, endA1, gyrA96, thl-1, hsdR17, supE44, relA1, lac[F’ proAB lacIq lacZ ΔM15] Tn10 (Tcr)*	Parsek et al., 1997
MG4	*Δ(argF-lac)U169, zah-735::T10, recA56, srl::Tn10*	Parsek et al., 1997
***P. aeruginosa* strains**	
PAO1	Wild type	Holloway et al., 1979
CW3	Δ*acp1*, derived from strain PAO1	This study
CW4	Δ*acp3*, derived from strain PAO1	This study
CW7	Δ*acp1*Δ*acp3*, derived from strain PAO1	This study
CW14	Δ*pqsR*, derived from strain PAO1	Gruber et al., 2016
CW16	Δ*lasR*, derived from strain PAO1	Gruber et al., 2016
CW17	Δ*rhlR*, derived from strain PAO1	Gruber et al., 2016
CW20	PAO1 with *acpP*-SPA in the chromosome	This study
CW21	PAO1 with *acp3*-SPA in the chromosome	This study
CW22	PAO1 with *acp1*-SPA in the chromosome	This study
CW26	Δ*katA*, derived from strain Δ*acp3*	This study
CW27	Δ*katA*, derived from strain Δ*acp1*Δ*acp3*	This study
CW28	Δ*katA*, derived from strain PAO1	This study
**Plasmids**		
pEX18ApGW	Suicide vector for *Pa*	Choi and Schweizer, 2005
pEX18ApGW-*acp1*	*acp1* deletion suicide vector	This study
pEX18ApGW-*acp3*	*acp3* deletion suicide vector	This study
pEX18ApGW-*pqsR*	*pqsR* deletion suicide vector	This study
pEX18ApGW-*lasR*	*lasR* deletion suicide vector	This study
pEX18ApGW-*rhlR*	*rhlR* deletion suicide vector	This study
pEX18ApGW-*katA*	*katA* deletion suicide vector	This study
pEX-HTB	*tacp*-6His on pEX1.8	This study
pEX-HTB-Acp1	Acp1 expression vector derived from pEX-HTB	This study
pEX-HTB-Acp3	Acp3 expression vector derived from pEX-HTB	This study
pEX-HTB-AcpP	AcpP expression vector derived from pEX-HTB	This study
pEX-HTB-KatA	KatA expression vector derived from pEX-HTB	This study
pECP61.5	*rhlA’-lacZ* transcriptional fusion; *tacp-rhlR*	de Lorenzo and Timmis, 1994
pKDT17	*lasB’-lacZ* transcriptional fusion; *tacp-lasR*	de Lorenzo and Timmis, 1994

To generate expression constructs of *acp1*, *acp3*, *acpP*, *E. coli acp*, and *katA*, the respective genes were amplified and cloned into pEX-HTB vector with restriction and ligation. pEX-HTB is derived from pEX1.8, and encodes a 6x-His tag within its multiple cloning site. All plasmids were confirmed by sequencing. For plasmid maintenance in *E. coli* cultures, the growth medium was supplemented with 50 μg/ml carbenicillin, 30 μg/ml kanamycin, or 30 μg/ml gentamycin. For *P. aeruginosa* cultures, 200 μg/ml carbenicillin or 30 μg/ml gentamycin were used, unless otherwise stated.

### Generation of *acp* Mutant Strains

Unmarked deletion mutants of *P. aeruginosa* were generated using a published method (Choi and Schweizer, 2005). Briefly, the target gene was replaced by a gentamycin (Gm^r^) resistance cassette through homologous recombination, followed by removal of the cassette by expression of Flp recombinase from pFLP2 and counter selection with 10% sucrose. All mutants were confirmed by PCR using appropriate flanking primers (**Supplementary Table S1**) and DNA sequencing. The deleted DNA fragments corresponded to amino acids 5–79 for Acp1 (94% of the protein sequence) and 7–45 for Acp3 (48% of the protein sequence). For *katA*, the deleted DNA corresponded to amino acids 21–448 (88% of the protein sequence).

### Quantitative RT-PCR of *acp* Transcripts

Transcript levels of *acp* genes were measured by real-time PCR using oligonucleotide primers designed with DNAMAN 7.0 software (Lynnon Cooperation). All primer pairs generated one specific product. The expression of gene *rpoD* was used as the housekeeping control. Cells were harvested at OD_600_ 0.5, 2.0, and 2.6, corresponding to the log, early stationary, and stationary phases, respectively. Total RNA was isolated using RNAqueous kit (Ambion), and genomic DNA was removed by precipitation with LiCl, followed by DNase treatment. cDNA was generated using the High Capability RNA-to-cDNA kit (Ambion), with 1 μg total RNA per reaction. Real-time quantitative PCR was performed in a 96-well plate, using the SensiFAST SYBR^®^ & Fluorescein kit (Bioline). The reaction mixture contained 500 nM gene-specific primers. Amplification and detection were performed on a Bio-Rad MyiQ single-color real time detection system. Experiments were performed in triplicate for each cDNA preparation. No-reverse transcriptase and no-template controls were included for each assay. Data were analyzed by MyiQ software, and the critical threshold cycle (*C*_T_) was set automatically. The amounts of *acp* gene mRNA were normalized against *rpoD* gene using the ΔΔC_T_ method (Rao et al., 2013).

### Protein Expression Profiling

The expression patterns of ACP proteins in *P. aeruginosa* were measured by western blotting. Overnight cultures were used to inoculate 100 ml fresh LB broth with an initial OD_600_ of 0.05, and incubated at 37°C with shaking. Samples were taken every 2 h, and cells lysed in B-PER reagent (Fisher). The total protein concentration was determined by Bradford assay. Fifty micrograms of total protein of the whole cell lysates was then separated by SDS-PAGE on 4–20% precast polyacrylamide gels and transferred to a PVDF membrane. SPA-tagged ACP proteins were detected by monoclonal anti-flag M2 (1:5000) (Sigma) and goat anti-mouse IgG-HRP (1:10,000) (Santa Cruz). Images were quantified by the software ImageJ (Schneider et al., 2012).

### Assays for Virulence Phenotypes

For static biofilm formation, cultures of early log phase were diluted into fresh LB to OD_600_ 0.05 and dispensed to a 96-well plate with 100 μl per well. The plate was covered with a transferable solid-phase (TSP) pegged-lid (Nunc) and incubated at 37°C for 18 h without shaking. Biofilms that formed on the pegged lid were washed twice by submerging the lid into a basin of sterilized water and then stained with 0.1% crystal violet (CV) in a new 96-well plate for 15 min with gentle shaking. Excess CV was washed off with water and then bound CV was eluted from the biofilms into 150 μl of 95% ethanol and quantitated spectrophotometrically at 600 nm using a Biotek Synergy HT plate reader.

### Tandem Affinity Purification

To construct strains expressing sequential peptide affinity (SPA)-tagged ACPs, a 250-bp DNA fragment encoding a calmodulin-binding peptide and 3× FLAG tag separated by a TEV cleavage site (Babu et al., 2009) was synthesized (Genewiz *Inc.*) and fused with a Gm^r^ cassette by overlapping PCR, generating a SPA-Gm^r^ fragment. Gene-specific upstream and downstream fragments were fused with this SPA-Gm^r^ fragment by overlapping PCR and cloned into pEX18ApGw to generate pEX18ApGw-*acp1/3/P*-SPA. Strains with C-terminal SPA-tagged *acp* were screened and confirmed by PCR and sequencing (Choi and Schweizer, 2005). SPA-tagged ACP proteins were detected by monoclonal anti-Flag M2 (1:5000) (Sigma) and goat anti-mouse IgG-HRP (1:10,000) (Santa Cruz).

Sequential peptide affinity purification was performed as described previously (Babu et al., 2009). Briefly, SPA-tagged strains and wild-type strain PAO1 were grown in 1 l of Terrific Broth in a 2-l flask at 37°C for 12 h with vigorous shaking. Cell pellets from stationary-phase cultures were harvested and lysed by sonication on ice. The cell lysates were treated with universal nuclease (Pierce) to remove nucleic acids and supplemented with protease inhibitor cocktail (Roche) to protect proteins from hydrolysis. The supernatant was mixed with magnetic beads conjugated with anti-Flag M2 antibody (Sigma) and incubated overnight on a rotary mixer at 4°C. Triton X-100 (0.1%) was added to prevent non-specific binding. The magnetic beads were collected by placing the tube in a magnetic rack, washed until the OD_280_ of flow-through was lower than 0.05, and re-suspended in 200 μl TEV cleavage buffer. TEV protease (40 μl of 1.8 mg/ml) was added and incubated overnight at 4°C with mixing. After the TEV cleavage step was complete, the magnetic beads were collected and the supernatant was transferred to a new tube, to which 400 μl of calmodulin-binding buffer and 1.6 μl of 1 M CaCl_2_ were added. The solution was then added to calmodulin Sepharose beads (GE Healthcare) and incubated for 3 h at 4°C with gentle mixing. The beads were washed four times with 1 ml of calmodulin-wash buffer (with EGTA). The bound proteins were eluted in 300-μl elution buffer. The target proteins were analyzed by MALDI-TOF mass spectrometry. The entire experiment was repeated once and only proteins identified in both experiments are reported.

### Reverse Pull-Down Assay

A His-KatA expression plasmid, pEXHTB-*katA*, and pEXHTB empty vector control were each transformed into strain CW21 (PAO1 with Acp3-FLAG). Cell pellets were harvested from 1 l of Terrific Broth after 12-h growth and cell lysates were prepared as described above. His-tagged proteins were purified by affinity chromatography using Ni^2+^-NTA agarose (Fisher). Flag-tagged-Acp3 in the elution was detected by western blotting with anti-Flag M2 antibody. Anti-ScKatA antibody was used to detect *P. aeruginosa* KatA (Shin et al., 2008).

### Determination of H_2_O_2_ Sensitivity

The sensitivity of different strains against hydrogen peroxide was determined by the microbroth dilution method in LB. One hundred microliters of a twofold serially diluted H_2_O_2_ solution was aliquoted to a 96-well plate. Cultures of early log phase were diluted in fresh LB to OD_600_ of 0.01 and mixed with H_2_O_2_ solution in equal volume. After incubation for 10 h at 37°C, cell density was measured at OD_600_. To further address the relationship between ACP and H_2_O_2_ resistance, pEX-HTB-derived ACP-expressing plasmids were introduced back into the Δ*acp1*/Δ*acp3* double mutant. Cultures were incubated in LB supplemented with 2 mM H_2_O_2_ at 37°C with vigorous shaking, and OD_600_ was measured after 5 h.

### Catalase Activity Assay

Catalase activity was measured as described previously (Ou and Wolff, 1996). A standard curve was established by mixing serially diluted H_2_O_2_ with FOX 1 reagent (250 μM ammonium ferrous sulfate, 100 μM xylenol orange, 0.1 M sorbitol, and 25 mM H_2_SO_4_). Cell pellets from 24-h cultures were washed once by PBS and lysed in B-PER reagent (Fisher). After centrifugation, the total protein in the supernatant was determined by Bradford assay. Ten microliters of supernatant was mixed with 990 μl of 200 μM H_2_O_2_ in 50 mM potassium phosphate (pH 7.0) and incubated for 1–30 min at room temperature. Fifty microliters of the mixture was then reacted with 950 μl of FOX 1 reagent at room temperature for 30 min. The reaction mixture was centrifuged and the absorbance of the resultant supernatant was measured at 560 nm. The concentration of remaining H_2_O_2_ was calculated based on the standard curve. The catalase unit activity was defined as the decomposition of 1 μmol of H_2_O_2_ in 1 min by 1 mg of total protein. Three independent experiments were performed. For comparison between replicates, relative catalase activity was calculated.

### Measurement of Intracellular ROS Level

Cell pellets from 10 ml of overnight cultures of wild-type strain and *acp* mutants were harvested, washed, and suspended in 1 ml of PBS. Cell suspensions were treated with 20 μM of 2′,7′-dichlorofluorescein diacetate (DCFH_2_, dissolved in DMSO) at 37°C for 20 min in the dark. Cells treated with an equal volume of DMSO were used as a negative control. The treated cells were washed once and suspended in 1 ml PBS. Two hundred microliters of suspension was transferred to a black opaque 96-well plate and fluorescence was measured using a BioTek microplate reader at 535 nm emission with excitation at 485 nm. The fluorescence data were normalized to the cell density of each sample. Three independent experiments were performed, and relative fluorescence was calculated by normalization to the wild-type strain for each experiment.

### Mouse Lung Infection Assay

Cells from overnight cultures of the *P. aeruginosa* wild-type strain PAO1 or isogenic *acp* mutant stains were harvested by centrifugation, washed twice and suspended in sterile PBS to 1 × 10^8^ CFU per 40 μl. Female C57Bl/6J mice (6–8 weeks old, Jackson Laboratories) were inoculated with 40-μl culture *via* oropharyngeal aspiration under anesthesia with isofluorane. Each treatment group contained 10 mice. At 48 h post infection, lungs were harvested, weighed, and homogenized in 1 ml of sterile PBS for bacterial enumeration. *P. aeruginosa* were grown and counted on PIA. The data were analyzed using a Kruskal–Wallis test with *post hoc* Dunn’s multiple-comparison tests to compare the bacterial loads among different groups. All procedures were in accordance with a protocol approved by the MUSC Institutional Animal Care and Use Committee.

## Results

### Expression of Acp3 Peaks in the Early Stationary Phase

**FIGURE 1:**
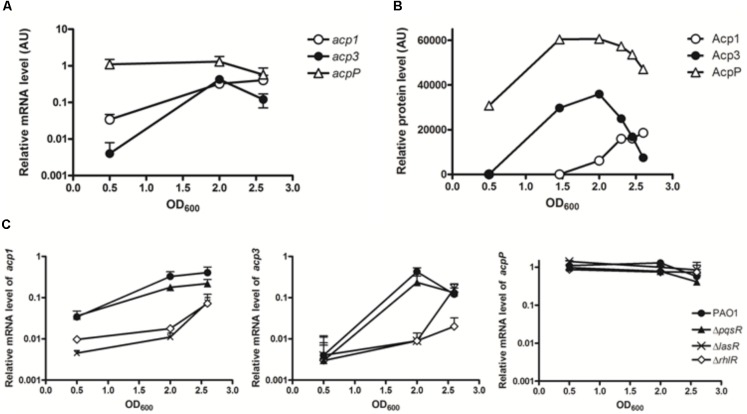
Expression profiles of the three *acp* genes in PAO1. **(A)** Transcripts levels were measured by qRT-PCR in log phase (OD_600_ 0.5), early stationary phase (OD_600_ 2.0), and stationary phase (OD_600_ 2.6). **(B)** Expression patterns of three ACP proteins in PAO1 were determined by western blotting with anti-Flag antibody. Samples were taken every 2 h post of inoculation. The immunoblot image was quantified with software ImageJ. **(C)** Regulatory connection between Acp1 and Acp3 and quorum sensing. Levels of *acp1* and *acp3* transcripts are lowered in the QS-deficient mutants, Δ*lasR* and Δ*rhlR*, but not Δ*pqsR*. *acp1*, left panel; *acp3*, middle panel; *acpP*, right panel.

As a preliminary step to investigate the function of Acp3, we used qRT-PCR to quantify the levels of all three *acp* transcripts in log phase (OD_600_ 0.5), early stationary phase (OD_600_ 2.0), and stationary phase (OD_600_ 2.6). The basal levels of the three transcripts were very different in log phase, with *acp3* being the least abundant and *acpP* the most abundant (**Figure 1A**). Transcript levels of *acp1* and *acp3* increased 9.6-fold and 106-fold, respectively, from log phase to early stationary phase. The level of *acp3* transcript then decreased in stationary phase, whereas that of *acp1* transcript level remained high. By contrast, the level of *acpP* expression remained high throughout, consistent with the expression profile of other known housekeeping *acpP* genes (Zhang et al., 2002).

To determine the endogenous protein levels of the three ACPs, we constructed strains of PAO1 in which the proteins were expressed as fusions with a SPA tag at the C terminus. The SPA tag contains a calmodulin-binding peptide and three FLAG domains. Semi-quantitative western blotting with an anti-Flag M2 antibody was used to measure protein levels at different growth stages. Commensurate with its function in membrane FAS, AcpP was constitutively expressed and also exhibited the highest expression level of the three ACPs (**Figure 1B**). The protein level of Acp3 mirrored its transcriptional pattern, indicating that acp3 expression is mainly regulated transcriptionally. The protein level of Acp1 was the lowest of the three ACPs in log phase, but increased as growth progressed to the stationary phase, which suggests it plays a role during late-stage growth.

### Transcription of *acp3* Is Regulated by QS Regulators

Previous microarray data have indicated that expression of *acp1* and *acp3*, but not *acpP*, are under the control of QS signals in *P. aeruginosa* (Hentzer et al., 2003; Schuster et al., 2003; Wagner et al., 2003). By contrast, a separate study using RNA-seq did not show activation of *acp3* expression by QS (Chugani et al., 2012). To determine whether the transcription of *acp1* and *acp3* is regulated by QS, we measured their respective transcript levels in the QS deletion mutants Δ*lasR*, Δ*rhlR*, and Δ*pqsR* (Gruber et al., 2016). Significantly reduced levels were observed for both *acp1* and *acp3* in the Δ*lasR* and Δ*rhlR* mutants, with at least a 10-fold decrease at an OD of 2.0 for both, but were unchanged in the Δ*pqsR* mutant (**Figure 1C**). Thus, it appears that expression of *acp1* and *acp3* are regulated by both LasR and RhlR systems, but not PqsR. By contrast, the level of the *acpP* transcript was unaffected in all QS mutants.

### Deletion of *acp3* Increases Bacterial Persistence of *P. aeruginosa* in the Murine Model

**FIGURE 2:**
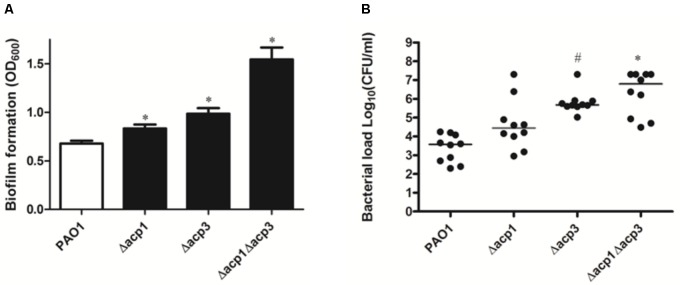
Deletion of *acp3* increases biofilm formation and persistence of *P. aeruginosa* PAO1 in a mouse lung infection model. **(A)** Biofilm formation increased in *acp* mutants. Biofilm formation by PAO1 and *acp* mutants was determined using a pegged lid 96-well plate. Data are presented as means ± SEs from three independent experiments. Student’s *t*-test was performed between PAO1 and *acp* mutant. ^∗^ indicates *p* < 0.01. **(B)** Determination of pathogenicity of *acp* mutants *in vivo*. Overnight cultures of wild-type and *acp* mutants were used to infect mice lungs. Forty-eight hours post inoculation, mice were sacrificed, lungs were removed and homogenized, and bacteria were counted on a PIA plate. Two independent experiments were performed. Data are presented as log (CFU/ml) analyzed using a Kruskal–Wallis test with *post hoc* Dunn’s multiple-comparison tests among different strains. Short bars within the data indicate the median CFU/ml. Significant *p* values are indicated by ^#^*p* < 0.05 and ^∗^*p* < 0.01.

Next, we investigated whether deletion of *acp3* had any effect on biofilm formation or *in vivo* pathogenicity. Compared to wild type, strains with single deletions of *acp1* or *acp3* enhanced static biofilm formation, but the effect was more pronounced in the double mutant (**Figure 2A**). This suggests that Acp1 and Acp3 each contribute to the virulence of *P. aeruginosa*. Additionally, all *acp* deletion strains exhibited higher bacterial loads in the lungs of C57Bl/6J mice, suggesting higher persistence *in vivo*, although the difference between the *acp1* mutant and PAO1 was not significant (**Figure 2B**). The strongest effect was observed with the *acp1/acp3* double mutant, for which the average colony-forming units (CFU) increased approximately three log units. Overall, these data suggest that deletion of *acp3* increases the pathogenicity of *P. aeruginosa*.

### Protein-Binding Partners of *P. aeruginosa* ACPs

To probe the physiological role of Acp3 further, we used tandem affinity purification (TAP) to identify its protein-binding partners, and alongside, the same for Acp1 and AcpP. Proteins identified only in strains expressing SPA-tagged ACPs but not PAO1 were considered potential ACP-binding partners. For the most part, the three ACPs have different sets of binding partners, commensurate with distinct physiological roles for these proteins. For AcpP, 10 protein candidates were identified (**Table 2**), including FabG, FabZ, FabA, GlmU, IscS, and LpxD, which are known to bind *E. coli* ACP (Butland et al., 2005). Their identification in *P. aeruginosa* provides further evidence that AcpP is functionally equivalent to *E. coli* ACP (Ma et al., 2017). Interactions with the Type II FAS enzymes FabG, FabZ, and FabA are consistent with a role for AcpP in fatty acid biosynthesis. New binding partners of AcpP were also found compared to those known for *E. coli* ACP, including CobL, LipB, SpeE2, and AlgB. One interaction that was expected but not observed was that between AcpP and SpoT, because these are known to form a complex in *P. aeruginosa* (Battesti and Bouveret, 2009).

**Table 2:** Binding partners of ACPs in *P. aeruginosa* identified by tandem affinity purification.

Locus	Protein	Function	SC^a^	SC	Abundance^b^	*p*-Value^c^
**AcpP**			**PAO1**	**CW20**		
PA2967	FabG	3-Oxo-acyl-ACP reductase	0	174	340.24	2.14E-54
PA3645	FabZ	3-Hydroxyacyl-ACP dehydratase	0	53	158.08	1.02E-17
PA2907	CobL	Precorrin-6YC(5,15)-methyltransferase	0	121	137.18	2.31E-38
PA5552	GlmU	Bifunctional enzyme of LPS/peptidoglycan synthesis	0	119	121.87	9.31E-38
PA1610	FabA	3-Hydroxydecanoyl-ACP dehydratase	0	22	58	3.35E-08
PA3814	IscS	Cysteine desulfurase	0	24	26.88	8.03E-09
PA3997	LipB	Octanoyltransferase	0	6	12.58	3.93E-03
PA4774	SpeE2	Spermidine synthase 2	0	6	7.59	3.93E-03
PA3646	LpxD	UDP-3-*O*-acylglucosamine N-acyltransferase	0	5	6.91	8.47E-03
PA5483	AlgB	Alginate biosynthesis transcriptional regulator	0	6	6.09	0.00393
**Acp1**			**PAO1**	**CW22**		
PA3831	PepA	Cytosol aminopeptidase	0	49	46	1.70E-16
PA3583	GlpR	Glycerol-3-phosphate regulon repressor	0	23	41	1.64E-08
PA2967	FabG	3-Oxo-acyl-ACP reductase	0	8	15.6	8.68E-04
PA4277	Tuf1	Elongation factor Tu	0	13	15	2.19E-05
**Acp3**			**PAO1**	**CW21**		
PA3814	IscS	Cysteine desulfurase	0	69	77.29	1.37E-22
PA3333	FabH2	3-Oxo-acyl-ACP synthase	0	49	72.09	1.70E-16
PA4236	KatA	Catalase	0	64	57.6	4.55E-21
PA5172	ArcB	Ornithine carbamoyltransferase	0	30	39.39	1.13E-10
PA4764	Fur	Ferric uptake regulation protein	0	8	26.27	8.69E-04
PA2967	FabG	3-Oxo-acyl-ACP reductase	0	11	21.51	9.43E-05
PA1588	SucC	Succinyl-CoA ligase	0	11	13.25	9.43E-05
PA4740	Pnp	Polyribonucleotide nucleotidyltransferase	0	17	11.27	1.21E-06
PA0999	PqsD	PQS biosynthetic protein	0	8	11	8.69E-04
PA5552	GlmU	Bifunctional protein	0	7	7.17	1.84E-03

*^a^SC is the summed number of spectral counts (i.e., MS/MS scans) assigned to one identified protein, used as a semi-quantitative index for protein abundance.*

*^b^Abundance = SC × 50 (kDa)/protein size (kDa). Proteins are listed with abundance above 5.*

*^c^p-Value less than 0.05 indicates significant change. The *p* value is derived by the G-test (Zhou et al., 2010).*

Only four binding partners of Acp1 were identified: PepA, GlpR, Tuf1, and FabG (**Table 2**). PepA is a leucine aminopeptidase that is homologous to *E. coli* PepA (Woolwine and Wozniak, 1999), GlpR is annotated as a repressor of the glycerol-3-phosphate regulon and Tuf1 as the elongation factor Tu (Winsor et al., 2016). One reason for there being fewer binding partners for Acp1 compared to AcpP or Acp3 might be the relatively lower expression of the protein at selected sampling time of 12 h (see **Figure 1B**).

Ten protein-binding partners of Acp3 were identified and most are enzymes of various functions (**Table 2**). The observed binding with IscS, GlmU, and FabG is in common with AcpP. Of the other proteins identified, novel binding partners include the 3-oxo-acyl-ACP synthase, FabH2 (PA3333), and also PqsD. *fabH2* is located within one putative operon with *acp3* (PA3334) and is of unknown function, in contrast to FabH (PA5814), which initiates fatty acid biosynthesis in *P. aeruginosa* (Zhang and Rock, 2012). PqsD is essential for the production of the quinolones DHQ and alkyl quinolones (Zhang et al., 2008). Additional partners are ArcB, an ornithine carbamoyltransferase, SucC, which is involved in the biosynthesis of succinate (Kapatral et al., 2000), and Fur, which is a ferric uptake regulator (Ochsner et al., 1995). Finally, KatA, which is the major catalase in *P. aeruginosa*, was identified as a binding partner of Acp3 and this was investigated further (see below).

### Interaction of KatA and Acp3 by Pull-Down Assay

**FIGURE 3:**
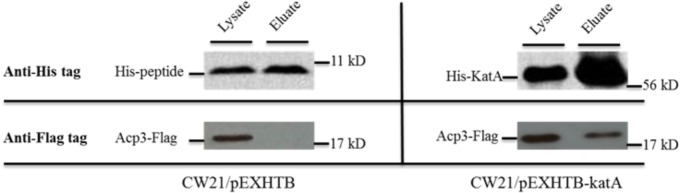
Binding of Acp3 and KatA by reverse pull-down assay. His-KatA and Acp3-FLAG were expressed in PAO1. Acp3 co-purified with His-tagged KatA after nickel-affinity chromatography, as detected by immunoblotting with anti-FLAG M2 antibody. Empty vector (pEXHTB) is a negative control. The full image of the immunoblots are shown in **Supplementary Figure S1**.

One of the most intriguing binding hits for Acp3 was KatA, which is the major catalase in *P. aeruginosa* and is required for resistance to peroxide and osmotic stress (Lee et al., 2005). To confirm that Acp3 and KatA interact with each other, we performed a reverse pull-down assay in which the pEXHTB plasmid expressing His-tagged KatA was transformed into PAO1 expressing FLAG-tagged Acp3 (CW21/pEXHTB-katA). The immunoblot shows that the FLAG-tagged Acp3 co-eluted with His-tagged KatA from the Ni^2+^-affinity column (**Figure 3**, right panel). The same strain transformed with vector expressing just the His tag was used as a comparison control (CW21/pEXHTB), and no Acp3 was detected in the eluate (**Figure 3**, left panel). This experiment confirms that Acp3 binds KatA specifically.

### *acp3* Mutant Is More Resistant to Hydrogen Peroxide

**FIGURE 4:**
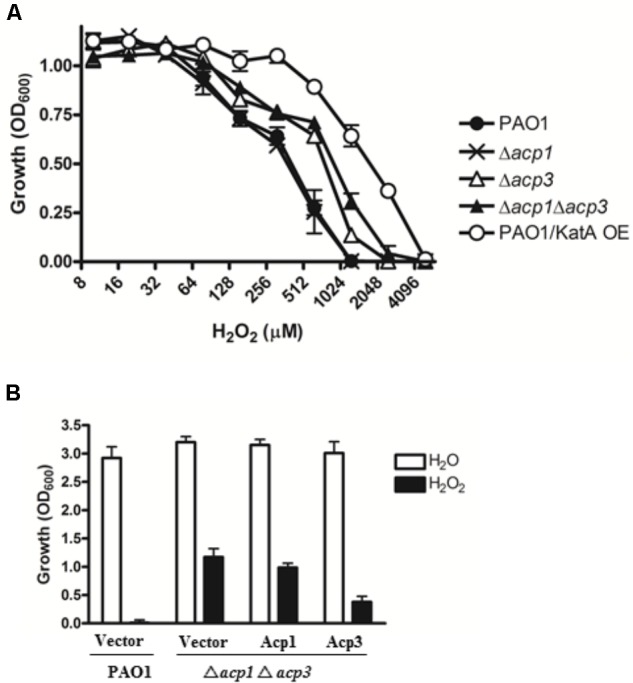
The effect of *acp* gene deletion or overexpression on *P. aeruginosa* resistance to H_2_O_2_. **(A)** Δ*acp3* mutant and Δ*acp1*Δ*acp3* double mutant were more resistant to H_2_O_2_ killing than PAO1 and the Δ*acp1* mutant. PAO1 overexpressing KatA (KatA OE) was used as a positive control. **(B)** Effect of ACP overexpression on resistance of *P. aeruginosa* to H_2_O_2_ (2 mM). Complementation of the Δ*acp1*Δ*acp3* double deletion mutant with a plasmid expressing Acp3 increased susceptibility to H_2_O_2_. Data are presented as mean ± SEs from three independent determinations.

One reason for the higher persistence of the *acp3* mutant in the mouse infection model could be increased tolerance to reaction oxygen species (ROS) related to the binding of Acp3 with KatA and potential influence on its activity. To examine this, we determined the H_2_O_2_ resistance of the *acp* mutants. Both the Δ*acp3* and Δ*acp1*/Δ*acp3* mutants exhibited a twofold higher resistance to killing by H_2_O_2_, compared with PAO1 (**Figure 4A**). The Δ*acp1* mutant exhibited similar susceptibility as wild type. As a positive control, overexpression of KatA increased resistance in PAO1 by fourfold. To confirm the effect of *acp* deletion on H_2_O_2_ resistance, we complemented the Δ*acp1*/Δ*acp3* mutant strain with plasmids expressing Acp1 or Acp3. When Acp3 was expressed, susceptibility of the strain to H_2_O_2_ was increased, whereas expression of Acp1 had little effect compared to the vector control (**Figure 4B**). These data show that the presence of Acp3 increases susceptibility of *P. aeruginosa* to H_2_O_2_.

### Acp3 Represses the Catalase Activity of KatA

**FIGURE 5:**
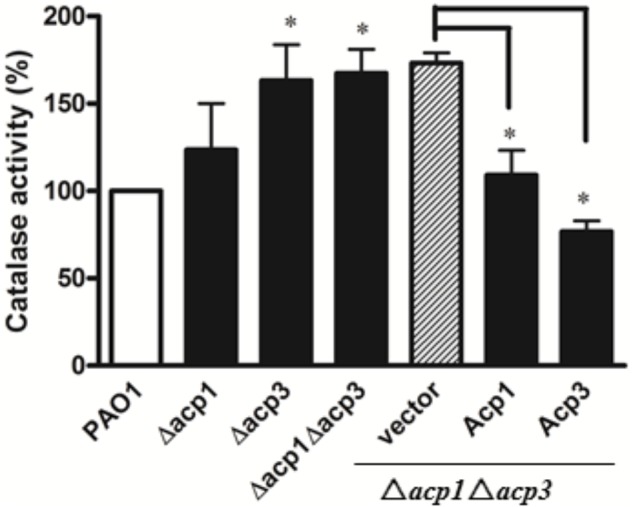
The absence of Acp3 increases the catalase activity of *P. aeruginosa*. Δ*acp3* and Δ*acp1*Δ*acp3* mutants exhibited higher catalase activity than wild-type PAO1. Catalase activity reduced after plasmids expressing Acp1 and Acp3 were introduced back into the Δ*acp1*Δ*acp3* mutant. Data are presented as mean ± SEs from three independent experiments. Student’s *t*-test was performed between PAO1 and *acp* mutants. ^∗^ indicates *p* < 0.01.

Since catalases play a major role in protecting bacteria against ROS *via* decomposition of H_2_O_2_ (Mishra and Imlay, 2012), we measured the catalase activity of the *acp1* and *acp3* deletion strains by the ferrous oxidation xylenol orange (FOX) assay (Ou and Wolff, 1996). Compared with the wild-type PAO1 strain, deletion of *acp3* enhanced the catalase activity of *P. aeruginosa* by 50%, with a lesser increase observed in the *acp1* deletion strain (**Figure 5**). Complementation of Acp3 in the Δ*acp1*/Δ*acp3* double mutants reduced catalase activity back to wild-type levels. These data are consistent with Acp3 acting as a repressor of KatA activity.

**FIGURE 6:**
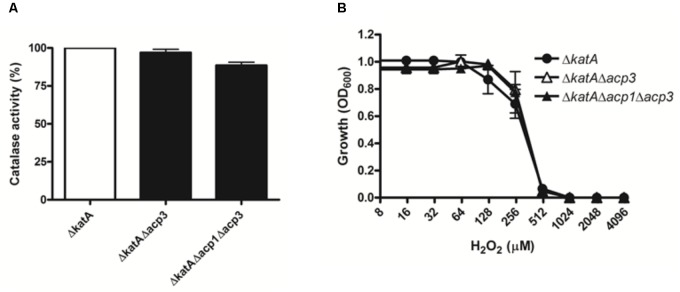
The H_2_O_2_ resistance of *acp3* mutant is mediated by KatA. In contrast to the effect of *acp3* deletion on catalase activity and susceptibility to H_2_O_2_ in the wild-type background, Δ*katA*, Δ*katA*Δ*acp3*, and Δ*katA*Δ*acp1*Δ*acp3* mutant strains exhibited similar **(A)** Catalase activity and **(B)** resistance against H_2_O_2_, suggesting that the increased catalase activity and resistance to H_2_O_2_ in the Δ*acp3* strain is dependent on KatA. Data are presented as mean ± SEs from three independent experiments.

To explore the functional relationship between Acp3 and KatA further, we deleted the *katA* gene in wild-type and *acp* mutants. Deletion of *katA* in PAO1 resulted in a nearly 300-fold reduction in catalase activity (data not shown). Importantly, there was no difference in catalase activity or resistance to H_2_O_2_ between the Δ*katA* and Δ*acp3/*Δ*katA* strains (**Figure 6**). This shows that the H_2_O_2_ resistance of the *acp3* mutant is mediated through KatA.

**FIGURE 7:**
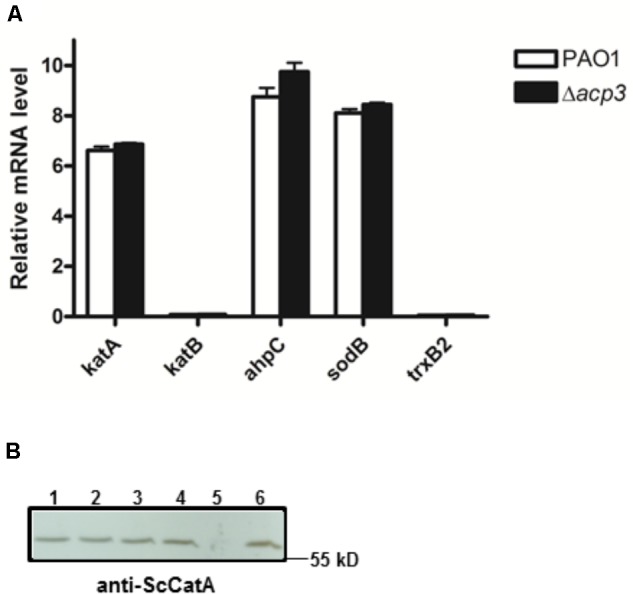
Deletion of *acp3* does not affect the transcript and protein levels of KatA in *P. aeruginosa*. **(A)** Transcriptional levels of selected genes involved in oxidative stress response were determined by qRT-PCR. Data are presented as mean ± SEs from three independent experiments. **(B)** Western blot analysis of KatA protein levels in PAO1 (lane 1), Δ*acp1* (lane 2), Δ*acp3* (lane 3), and Δ*acp1*Δ*acp3* (lane 4). The Δ*katA* strain (lane 5) and purified His-KatA (line 6) were used as negative and positive controls, respectively. Cells from overnight cultures were used to prepare total RNA and the total protein samples for the analyses.

To address whether the absence of Acp3 affects the expression level of *katA*, we measured the level of *katA* transcript in the *acp3* deletion strain, as well as for several genes that respond to oxidative stress, specifically *katB*, *ahpC*, *sodB*, and *trxB2* (Salunkhe et al., 2005). Compared to PAO1, deletion of *acp3* did not alter transcription levels of *katA* or any of the other selected genes (**Figure 7A**). Similarly, protein levels of KatA were the same in *acp1* or *acp3* deletion strains compared to PAO1 (**Figure 7B**). These data suggest that the activity of KatA is inhibited by a direct interaction with Acp3 and not by transcriptional or translational regulation.

### Deletion of *acp3* Reduces Endogenous ROS Levels

**FIGURE 8:**
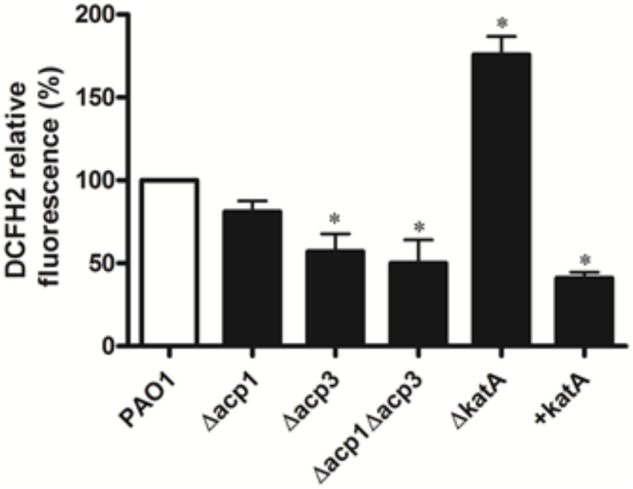
Deletion of *acp3* gene decreases endogenous ROS. ROS levels were measured by DCFH2 fluorescence. katA-deletion (ΔkatA) and KatA-overexpressing (+katA) strains were used as controls. Data are presented as mean ± SEs from three independent experiments. Student’s *t*-test was performed between PAO1 and deletion or over-expressing strains. ^∗^ indicates *p* < 0.01.

To determine whether inhibition of KatA activity by Acp3 correlates with levels of endogenous ROS, we used DCFH_2_, a non-specific fluorescence probe, to measure ROS levels in *P. aeruginosa* cells (Brömme et al., 2008). Significantly less ROS was detected in the Δ*acp3* mutant compared with wild type, commensurate with a higher level of KatA activity (**Figure 8**). In addition, the *katA* deletion mutant contained a much higher level of ROS and the KatA overexpression strain exhibited lower ROS. These data suggest that release of KatA inhibition by the *acp3* deletion decreases ROS levels in *P. aeruginosa*.

## Discussion

In addition to canonical ACPs that function during FAS, bacteria typically express other ACPs and in many cases, the functions of these homologues are unknown. Following a recent study in which AcpP was confirmed as the essential ACP involved in membrane FAS (Ma et al., 2017), we investigated Acp1 and Acp3 of *P. aeruginosa* in order to shed light on their physiological roles in this important pathogen. The major finding is that Acp3 impacts virulence and *in vivo* pathogenicity of *P. aeruginosa* and also appears to regulate the activity of the catalase KatA in a way that suggests involvement in the cellular response to oxidative stress.

In keeping with the idea that Acp1 or Acp3 have a role in pathogenesis, we first investigated whether their expression is regulated by QS. This is an important mechanism employed by bacterial populations to respond to environmental cues, such as nutrient restriction or the presence of antibiotics. In *P. aeruginosa*, QS governs biofilm formation, swarming motility, and production of virulence factors such as pyocyanin, elastases, and rhamnolipids (Venturi, 2006). A connection between ACPs and QS is logical because the synthesis of QS signals such as PQS and acyl homoserine lactones (HSLs) requires acyl ACP (Parsek et al., 1997; Raychaudhuri et al., 2005; Dulcey et al., 2013). Furthermore, the higher levels of both transcripts and proteins for ACPs observed during the transition of cell cultures from log to stationary phase could involve QS. Accordingly, we found that levels of *acp1* and *acp3* transcripts are significantly lower in *P. aeruginosa* strains lacking LasR or RhlR, which are the receptors for 3-oxo-C12-HSL and C4-HSL, respectively (Gambello and Iglewski, 1991; Ochsner and Reiser, 1995) and thus suggests that expression of Acp1 and Acp3 is governed by the QS machinery.

Our next step was to assess whether Acp1 or Acp3 plays a role in pathogenicity. We found that deletion of *acp1* or acp3 has no effect on *in vitro* virulence of *P. aeruginosa*, except for thicker biofilm. By contrast, when tested in a mouse infection model, a 1000-fold increase in bacterial load was detected for both *acp1* and *acp3* deletion mutants, indicating that both Acp1 and Acp3 play a role in *P. aeruginosa* pathogenicity *in vivo*. A previous study showed increased expression levels of both *acp1* and *acp3* genes in acute and chronic wound infection models in mice (Turner et al., 2014), suggesting that Acp1 and Acp3 are important for *P. aeruginosa* fitness in a disease environment *in vivo*. The different effects of *acp1* and *acp3* deletion on virulence *in vitro* versus *in vivo* may be due to the differences in the availability of nutrients and oxygen in the growth environment.

To probe their physiological roles further, we used TAP (Babu et al., 2009) to identify protein-binding partners of Acp1 and Acp3. The identification of several enzymes involved in fatty acid biosynthesis as binding partners of AcpP provided validation for the experiment. Acp1 and Acp3 also bound some enzymes of Type II FAS, but more so to proteins from other areas of metabolism. For instance, the top hit for Acp1 was PepA (also known as PhpA), which possesses aminopeptidase activity and whose disruption in an *algB* mutant increases alginate expression in *P. aeruginosa* (Woolwine and Wozniak, 1999). PepA also plays a role within the Xer recombinase system of *E. coli* (where it is called XerB) (Stirling et al., 1989) and it lowers expression of carbamoyl phosphate synthase by binding to the promoter region of the *carAB* operon in *E. coli* (Charlier et al., 1995). The association of PepA with Acp1 adds a further dimension to a protein that appears to function in several very different processes. Another binding hit for Acp1 is GlpR, which is a repressor of genes involved in glycerol metabolism (Schweizer and Po, 1996). Since genetic deletion of GlpR increases biofilm formation in *P. aeruginosa* (Scoffield and Silo-Suh, 2016), it is tempting to speculate that its association with Acp1 may affect virulence.

Among the proteins that bound Acp3 were IscS, FabH2, KatA, and ArcB. The breadth and diversity of these interactions hints at the versatility of Acp3 in its functions. The top hit was IscS, a cysteine desulfurase that provides sulfur for the biosynthesis of FeS clusters, thiamine, biotin, lipoic acid, modified nucleosides in tRNAs, and the molybdenum cofactor (Moco), among others (Hidese et al., 2011; Leimkuhler et al., 2017). Its interaction with Acp3 is not the first evidence of a functional association between ACPs and cysteine desulfurases. Using the same TAP approach, *E. coli* ACP was also found to bind IscS, with the interaction mediated by a disulfide bond between the 4-phosphopantetheine group and Cys328 of IscS (Gully et al., 2003). It remains to be established whether this group is also required for the interaction in *P. aeruginosa*.

Interactions between IscS and ACP also occur in humans, albeit indirectly. A multisubunit complex containing NFS1 (the name given to IscS in humans, which shares ∼60% amino acid sequence identity with *E. coli* IscS), the LYR protein ISD11 and acyl ACP assembles FeS clusters in mitochondria (Van Vranken et al., 2016). Within this complex, ACP interacts with the ISD11 subunit (Herrera et al., 2018), which in turn interacts with NSF1 (Cory et al., 2017). Such Acp–IscS interactions in other species supports the idea that Acp3 may have a role in assembly of FeS clusters in *P. aeruginosa*.

Another putative binding partner for Acp3 is ArcB, an ornithine carbamoyl transferase (OCT). This enzyme is part of the arginine deiminase system (ADS) found in bacteria that allows anaerobic growth on arginine. In *P. aeruginosa*, OCT is a dodecameric complex of identical protomers (Marcq et al., 1991) and is allosterically inhibited by spermidine and activated by AMP regulation (Tricot et al., 1993). There appears no involvement of ACP in the reaction mechanism (Tricot et al., 1998) and this putative interaction therefore requires further investigation.

The apparent promiscuity in binding of *P. aeruginosa* ACPs and especially those for Acp3 is mirrored by *E. coli* ACP, which binds to a number of protein factors outside of its canonical function in FAS, including IscS, MukB, YbgC, and SpoT (Gully et al., 2003; Gully and Bouveret, 2006). Of these, the interaction with SpoT has been examined in most detail (Battesti and Bouveret, 2006; Angelini et al., 2012). SpoT is a factor that changes its activity from (p)ppGpp hydrolysis to (p)ppGpp synthesis in response to starvation signals, leading to initiation of the stringent response. It has been postulated that the switch in its enzymatic activity toward synthesis may be governed by the binding of ACP to the C-terminal domain of SpoT (Battesti and Bouveret, 2006). Mutations that abrogate binding to SpoT mostly map to hydrophobic residues in helix II of ACP (Angelini et al., 2012) and this region of the protein also mediates interactions with its other protein partners, including FabI, AcpS, and YchM(Parris et al., 2000; Rafi et al., 2006; Babu et al., 2010). Each of the new potential binding partners revealed here for Acp1 and Acp3 offers opportunity to investigate the molecular details of these interactions in a similar way as ACP-SpoT, including establishing whether helix II is a common interface, determining whether an acylated 4-phosphopantetheine prosthetic group is involved in the binding, and understanding their physiological consequences in *P. aeruginosa*.

With this goal in mind, we decided to investigate further the interaction of Acp3 with KatA, the major catalase of *P. aeruginosa* (Hassett et al., 1992). *katA* is one of the oxidative stress response genes expressed during aerobic metabolism or in response to exogenous ROS, and its expression is controlled by the global transcriptional regulator OxyR (Heo et al., 2010). At first sight, the reason for an interaction between Acp3 and KatA is not obvious, but can be rationalized in the context of cellular responses of *P. aeruginosa* to oxidative stress. Under such conditions, KatA is upregulated in order to dismutate H_2_O_2_ and other ROS, and presumably is negatively regulated when ROS levels normalize. It appears that by binding to KatA, Acp3 acts as a negative regulator of its catalase activity. This is supported by the increased resistance of the *acp3* deletion strain to H_2_O_2_ (because KatA activity is constitutively higher without Acp3). Higher tolerance to ROS may also explain the increased persistence of the *acp3* deletion mutant *in vivo.* The inhibition of KatA by binding of Acp3 is reminiscent of the ACP-SpoT interaction and a further example of how ACPs can regulate enzyme activities.

It is interesting to note that the putative interaction between Acp3 and IscC mentioned above may also be related to oxidative stress. This is because a strain of *C. difficile* lacking IscS2 (the homologue of IscS) fails to grow when oxygen levels go above 2% (Giordano et al., 2018) suggesting it protects against oxidative stress. However, it needs to be established whether binding of Acp3 to IscS represses its cysteine desulfurase activity similar to KatA, or whether it enhances activity.

Why Acp3 regulates the activity of KatA is unknown. There are probably several potential reasons that could be invoked, but one intriguing possibility relates to the so-called “Insurance Hypothesis.” This is the diversification of phenotype in bacterial communities in response to harsh conditions (Boles et al., 2004). The increased expression of Acp3 prior to stationary phase may serve to reduce catalase activity and thereby increase ROS levels. The subsequent DNA damage and mutation would increase genotypic and phenotypic diversity within the biofilm (Boles et al., 2004), thus allowing the community to survive. While more work is needed to uncover its details, the observed binding of Acp3 to KatA reveals an additional mechanism of control within an already complex regulatory network for KatA and a potential role for this ACP in response to oxidative stress.

## Author Contributions

WC generated all strains for this work, performed the phenotype analyses and enzymatic assays *in vitro*, prepared the figures, and contributed to the writing of the manuscript. BW and JG performed the *in vivo* pathogenicity assays. Y-MZ conceived the study, directed the experiments, and helped draft the manuscript. CD contributed ideas to the project and helped draft the manuscript. All authors contributed to the analysis and interpretation of data. All authors approved the manuscript submitted.

## Conflict of Interest Statement

Y-MZ is currently employed by Jeneil Biotech Inc. The remaining authors declare that the research was conducted in the absence of any commercial or financial relationships that could be construed as a potential conflict of interest.
