# Supplementary material for: Acyl Carrier Protein 3 Is Involved in Oxidative Stress Response in Pseudomonas aeruginosa
Source: Front Microbiol. 2018 Sep 20;9:2244. doi: 10.3389/fmicb.2018.02244 (PMC6158461; doi:10.3389/fmicb.2018.02244)
Supplement: Supplementary file 1 [file Table_1.PDF]

Supplemental Table S1: Primers used in this study

Primers	Sequences	Use
acp1'UpF	TACAAAAAAGCAGGCTCAGCAGCGAAGCCTTCATG	to delete *acp1*
acp1'UpR	TCAGAGCGCTTTTGAAGCTAATTCGTCGATGTCGTCCATGCATTG	
acp1'DnF	AGGAACTTCAAGATCCCCAATTCGTGGTCATTCCCTGCGAAAC	
acp1'DnR	TACAAGAAAGCTGGGTATGCGGTCCTGGATATCATC	
acp3'UpF	TACAAAAAAGCAGGCTCAGTTCGACCTGCTGATGAC	to delete *acp3*
acp3'UpR	TCAGAGCGCTTTTGAAGCTAATTCGTTCCATGTCGTTGGGCATTG	
acp3'DnF	AGGAACTTCAAGATCCCCAATTCGGAAGCTGCGCAAGGAGTTC	
acp3'DnR	TACAAGAAAGCTGGGTATCGCCTCGAAGGTCATCAG	
rhlR'UpF	TACAAAAAAGCAGGCTAGGATGAACGGCAGGCAAC	to delete *rhlR*
rhlR'UpR	TCAGAGCGCTTTTGAAGCTAATTCGGATCGAGTTGCTGACCCAG	
rhlR'DnF	AGGAACTTCAAGATCCCCAATTCGGGGTATGACGCTGTCAAGC	
rhlR'DnR	TACAAGAAAGCTGGGTACGACCAGTTCGACAATGC	
lasR'UpF	TACAAAAAAGCAGGCTTCGCCGAACTGGAAAAGTGG	to delete *lasR*
lasR'UpR	TCAGAGCGCTTTTGAAGCTAATTCGGATATCCGGCACGTTGTACG	
lasR'DnF	AGGAACTTCAAGATCCCCAATTCGAAGGAAGTGTTGCAGTGGTG	
lasR'DnR	TACAAGAAAGCTGGGTGAACCCGGACCCTTGCTAG	
pqsR'UpF	TACAAAAAAGCAGGCTGGTGATGCGCAACGATACG	to delete *pqsR*
pqsR'UpR	TCAGAGCGCTTTTGAAGCTAATTCGCAACCGAGCATCGTCGAAAC	
pqsR'DnF	AGGAACTTCAAGATCCCCAATTCGTCAGGTTGCCGCTACTGTG	
pqsR'DnR	TACAAGAAAGCTGGGTTGAGCAAACCCCGGGGATC	
katA'UpF	TACAAAAAAGCAGGCTCTGGTTGATCGTCCTGTC	to delete *katA*
katA'UpR	TCAGAGCGCTTTTGAAGCTAATTCGGTTCTGGTTATCGACCACC	
katA'DnF	AGGAACTTCAAGATCCCCAATTCGCGATGTACCGGAACAGATC	
katA'DnR	TACAAGAAAGCTGGGTGGGTCTCCTCCTCCATCTC	
SCN-acp1F2	ATCAGCCATGGCCGTTACC	screen *acp1* deletion mutant
SCN-acp1R2	ATCGTCCATGCCGACGTTATC	
SCN-acp3F	TTTCAGCCATACCCTCGACC	screen *acp3* deletion mutant
SCN-acp3R	GTGTGGTTGAGGATGTCGTCG	
SCN-rhlRF	GGCATCCCTACCCTGATACTC	screen *rhlR* deletion mutant
SCN-rhlRR	TCGACCGAACACCTGCAG	
SCN-lasRF	CTGCGTGATGCTGCAACTG	screen *lasR* deletion mutant
SCN-lasRR	CGTCGATGACACTAACGTCC	
SCN-pqsRF	CTTCACCTACAACCTGGTCG	screen *pqsR* deletion mutant
SCN-pqsRR	GCGTCATAGTCGCTACACC	
SCN-katA-F	CAAGCTGTTCAACGACCTG	screen *katA* deletion mutant
SCN-katA-R	CTTCGAGCAGGAGGATACG	
1.8F	CGACATCATAACGGTTCTGGC	universal primer for pEX1.8
1.8R2	CCAGGCAAATTCTGTTTTATCAGACCGC	universal primer for pEX1.8
Flag-F2	TCCATGGAAAAGAGAAGATG	to clone Flag

Flag-R	AGTATAGGAACTTCAGAGC	sequence
Flag-acpP-UpF	TACAAAAAAGCAGGCTCATCGACACCGACATGACC	to label *acpP* with Flag in the chromosome
Flag-acpP-UpR	CGGCTATGAA ATTCTTTTTC CATCTTCTCT<br>TTTCCATGGATTGCTGGTGAGCAACGATG	
Flag-acpP-DnF	AGGAACTTCAAGATCCCCAATTCGTAAGTAGTCGTCGGATTTTCCG	
Flag-acpP-DnR	TACAAGAAAGCTGGGTCACGAAGAACGGCGAGATG	
Flag-acp1-UpF	TACAAAAAAGCAGGCTCGCGATGGCCAGGAGCTGTTC	to label *acp1* with Flag in the chromosome
Flag-acp1-UpR	CGGCTATGAA ATTCTTTTTC CATCTTCTCT<br>TTTCCATGGAGGTCGGCACGGCTTCCTCGA	
Flag-acp1-DnF	AGGAACTTCAAGATCCCCAATTCGTGACTCCCCCTGGACCACC	
Flag-acp1-DnR	TACAAGAAAGCTGGGTGCCAGATCGTCCATGCCGAC	
Flag-acp3-UpF	TACAAAAAAGCAGGCTCAGGCCAACACACGCATTCTCG	to label *acp3* with Flag in the chromosome
Flag-acp3-UpR	CGGCTATGAA ATTCTTTTTC CATCTTCTCT<br>TTTCCATGGAGGCGGCGCGGTGCTTCTCG	
Flag-acp3-DnF	AGGAACTTCAAGATCCCCAATTCGTGA GGCCAGCGAA<br>CGCCGGCGTT C	
Flag-acp3-DnR	TACAAGAAAGCTGGGTCCCAGCAGGTTGAACGGCAGCAG	
