# Supplementary material for: Acyl Carrier Protein 3 Is Involved in Oxidative Stress Response in Pseudomonas aeruginosa
Source: Front Microbiol. 2018 Sep 20;9:2244. doi: 10.3389/fmicb.2018.02244 (PMC6158461; doi:10.3389/fmicb.2018.02244)
Supplement: Supplementary file 2 [file Image_1.PDF]

Fig. S1. Full gel images used for Fig. 3 of the main manuscript

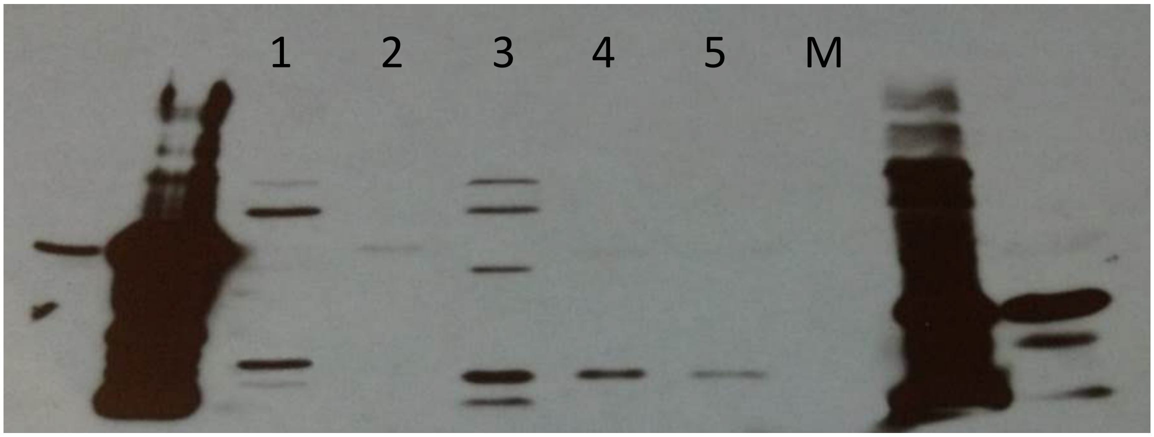

Western blotting for Acp3-KatA pull down with anti-Flag antibody.
Lane 1, lysate of CW21/pEXHTB; lane 2, eluate of CW21/pEXHTB; lane 3, lysate of CW21/pEXHTB-katA; lanes 4 and 5, eluate of CW21/pEXHTB-katA; lane 6, markers.

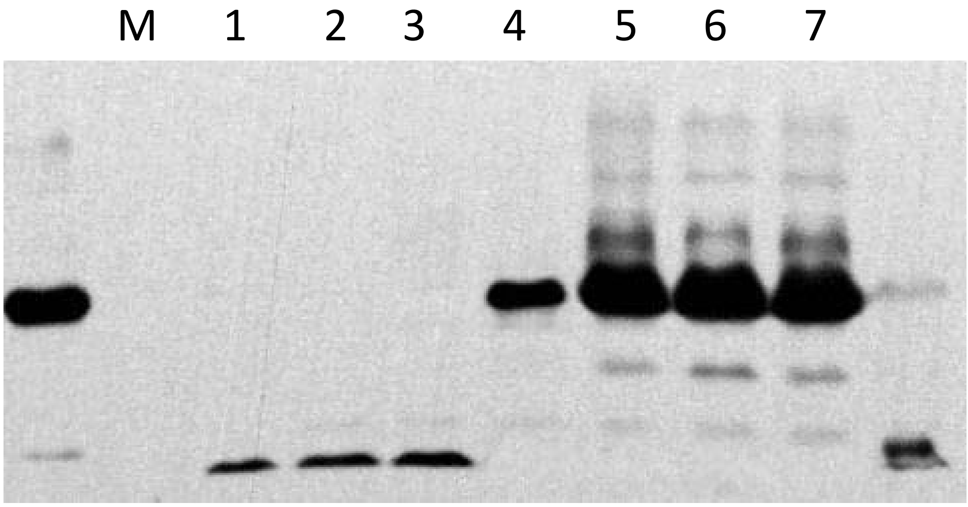

Western blotting for Acp3-KatA pull down with anti-His antibody.
Lane 1, lysate of CW21/pEXHTB; lane 2 and 3, eluate of CW21/pEXHTB; lane 4, lysate of CW21/pEXHTB-katA; lane 5-7 , eluate of CW21/pEXHTB-katA; lane 6, Marker.
